# Life Crafting as a Way to Find Purpose and Meaning in Life

**DOI:** 10.3389/fpsyg.2019.02778

**Published:** 2019-12-13

**Authors:** Michaéla C. Schippers, Niklas Ziegler

**Affiliations:** Department of Technology and Operations Management, Rotterdam School of Management, Erasmus University, Rotterdam, Netherlands

**Keywords:** life crafting, meaning in life, scalable life-crafting intervention, Ikigai, goal setting, positive psychology, well-being and happiness, self-concordance

## Abstract

Having a purpose in life is one of the most fundamental human needs. However, for most people, finding their purpose in life is not obvious. Modern life has a way of distracting people from their true goals and many people find it hard to define their purpose in life. Especially at younger ages, people are searching for meaning in life, but this has been found to be unrelated to actually finding meaning. Oftentimes, people experience pressure to have a “perfect” life and show the world how well they are doing, instead of following up on their deep-felt values and passions. Consequently, people may need a more structured way of finding meaning, e.g., *via* an intervention. In this paper, we discuss evidence-based ways of finding purpose, *via* a process that we call “life crafting.” This process fits within positive psychology and the salutogenesis framework – an approach focusing on factors that support human health and well-being, instead of factors that cause disease. This process ideally starts with an intervention that entails a combination of reflecting on one’s values, passions and goals, best possible self, goal attainment plans, and other positive psychology intervention techniques. Important elements of such an intervention are: (1) discovering values and passion, (2) reflecting on current and desired competencies and habits, (3) reflecting on present and future social life, (4) reflecting on a possible future career, (5) writing about the ideal future, (6) writing down specific goal attainment and “if-then” plans, and (7) making public commitments to the goals set. Prior research has shown that personal goal setting and goal attainment plans help people gain a direction or a sense of purpose in life. Research findings from the field of positive psychology, such as salutogenesis, implementation intentions, value congruence, broaden-and-build, and goal-setting literature, can help in building a comprehensive evidence-based life-crafting intervention. This intervention can aid individuals to find a purpose in life, while at the same time ensuring that they make concrete plans to work toward this purpose. The idea is that life crafting enables individuals to take control of their life in order to optimize performance and happiness.

*The best day of your life is the one on which you decide your life is your own. No apologies or excuses. No one to lean on, rely on, or blame. The gift is yours – it is an amazing journey – and you alone are responsible for the quality of it. This is the day your life really begins*.

—Bob Moawad

## Introduction

Whether you love him or hate him, Arnold Schwarzenegger is an example of a person who has been planning his life and setting goals throughout. Given that he came from a small town in Austria, the chances of him becoming the person he is today were very slim. Although even his parents thought that his ideas of becoming a great body builder were outrageous and his fellow cadets made fun of him when he put in extra hours of training while he was in the military, holding on to his vision and dreams paid off in the end (see Schwarzenegger and Hall, 2012). So even though it was not obvious that he would achieve the goals he had set for himself, he made a plan and stuck to his plan to achieve his goals.

Now consider this story: Brian is CEO of a large bank, and seems by all standards to be living a fulfilling live. Although he is overseeing 1,200 employees, earns a good salary, has a nice house at the beach, and a wife and kids, he feels very unhappy with his current life. One day he decides that he does not want to live this life anymore and quits his job. He becomes a consultant (and his wife divorces him) but still struggles to find his passion. As he knows that the job he is doing is not his passion, he starts exploring what he would like to do. Unfortunately, having done things for so long that have not brought him satisfaction, only status and money, he seems to have trouble connecting to his “inner self.” In his search for why he has ended up this way, he realizes that he has been living the life his father had in mind for him. This leads him to think that, if it had not been for his father, he would probably have studied psychology instead of management.

These two, seemingly unrelated anecdotes, tell something very important: no matter how successful a person is in life, self-endorsed goals will enhance well-being while the pursuit of heteronomous goals will not (for a review see Ryan and Deci, 2001). This is an important statement and key to self-determination theory (SDT, Ryan and Deci, 2000), a macro-theory of human motivation, stressing the importance of self-motivated and self-determined goals to guide behavior for well-being and happiness. Goal attainment from self-concordant goals, or goals that fulfill basic needs and are aligned with one’s values and passions, has been related to greater subjective well-being (Sheldon, 2002), higher vitality (Nix et al., 1999), higher levels of meaningfulness (McGregor and Little, 1998), and lower symptoms of depression (Sheldon and Kasser, 1998). Self-concordant goals satisfy basic psychological needs of autonomy, competence, and relatedness, key attributes of SDT (Ryan and Deci, 2001), and have been found to be important across cultures (see Sheldon et al., 2004). With an increasing number of young people experiencing mental health problems, increasing health care costs and an aging society, the interest in cost-effective behavioral interventions that can improve mental and physical health is burgeoning (e.g., Oettingen, 2012; Fulmer et al., 2018; Chan et al., 2019; Wilson et al., 2019; for reviews see Wilson, 2011; Walton, 2014). Especially promising is the research on the topic of meaning and purpose in life (Steger, 2012). People with a purpose in life are less likely to experience conflict when making health-related decisions and are more likely to self-regulate when making these decisions and consequently experience better (mental) health outcomes (Kang et al., 2019). Furthermore, having a purpose in life can aid in overcoming stress, depression, anxiety, and other psychological problems (see Kim et al., 2014; Freedland, 2019). Finally, purpose in life has been related to a decrease in mortality across all ages (Hill and Turiano, 2014). It thus appears that many benefits may be gained by enhancing meaning and purpose in life. However, even if people realize they are in need of a purpose, the search for meaning does not automatically lead to its presence, and people searching for meaning are no more or less likely to plan for and anticipate their future (Steger et al., 2008b). This somewhat counterintuitive finding, showing that among undergraduate students the search for meaning is even inversely related to presence of meaning, points to the fact that the strategies people use to find meaning may not be very effective (Steger et al., 2008b). Early in life, the search for meaning is not negatively related to well-being, but the relationship between search for meaning and well-being becomes increasingly negative in later life stages (Steger et al., 2009). This means that even if people search for meaning, they may not find it, unless they are prompted to do so in an evidence-based manner, e.g., *via* a positive psychology intervention. Especially adolescents and young adults should be stimulated to search for meaning in an organized manner in order to experience higher levels of well-being early in life so that they can be more likely to have an upward cycle of positive experiences. An intervention to bring about purpose in life may be a promising way to achieve this. Recent research suggests that interventions aimed at enhancing purpose in life can be particularly effective if they are done early on, during adolescence and/or as part of the curriculum in schools (Morisano et al., 2010; Bundick, 2011; Schippers et al., 2015).

These interventions address an important contemporary problem, as illustrated by the two anecdotes above, namely that, many people drift aimlessly through life or keep changing their goals, running around chasing “happiness” (Donaldson et al., 2015). Others, as in the example of Brian above, live the life that their parents or significant others have in mind for them (Kahl, 1953). Several authors have indeed noted that the role of parents in students’ study and career choices has been under-researched (Jodl et al., 2001; Taylor et al., 2004), but choosing one’s study and career path according to one’s own preferences is likely to be more satisfying than living the life that others have in mind for one. Recently, it has been noted that especially “socially prescribed” perfectionism where people try to live up to the standards of other and also seek their approval is related to burn-out, depression and a lack of experienced meaning (Suh et al., 2017; Garratt-Reed et al., 2018; Curran and Hill, 2019). In our society, education is highly valued, but less emphasis is placed on structured reflection about values, goals, and plans for what people want in life. Oftentimes, education fosters maladaptive forms of perfectionism, instead of adaptive forms (Suh et al., 2017). Even if parents and educators do ask children what they want to become when they grow up, this most important question is not addressed in a consistent way that helps them to make an informed choice (Rojewski, 2005). Parents and educators tend to look at the children’s competences, rather than what they want to become and what competences they would need to develop in order to become that person (Nurra and Oyserman, 2018). Consequently, many people only occupy themselves with the daily events in their lives, while others try to keep every aspect of their lives under control and live the life that others have in mind for them. Some have an idea of what they want but have not thought about it carefully. Others may have too many goals, or conflicting goals, which is also detrimental to health and well-being (Kelly et al., 2015). Finally, parents and others with the best of intentions sometimes have goals in mind for children to pursue (Williams et al., 2000; Tamis-LeMonda et al., 2008).

A study by Nurra and Oyserman (2018) showed that children that were guided to experience connection between their current and adult future self, worked more and attained better school grades than children guided to experience low connection. Importantly, this was moderated to the extent that children saw school as the path to one’s adult future self. It seems important that people formulate and think about their (ideal) future self and that the present and future self are connected, e.g., by means of a goal-setting intervention. Studies among students also showed the importance of goal congruence. For instance, Sheldon and Kasser (1998) found that although students with stronger social and self-regulatory skills made more progress in their goals, and goal progress predicted subjective well-being (SWB), while the increase in well-being depended on the level of goal-congruence. Similarly, Sheldon and Houser-Marko (2001) found that entering freshman students with self-concordant motivation had an upward spiral of goal-attainment, increased adjustment, self-concordance, higher ego development, and academic performance after the first year. This points to the importance of making sure people reflect on and develop self-concordant goals (Locke and Schippers, 2018). If people have not formulated their own goals, there is a chance that they will lose contact with their core values and passions,” (Seto and Schlegel, 2018) as was the case in the anecdote of Brian. It may even feel as if they are living someone else’s life. For several reasons, it is important that people take matters into their own hands and reflect on and formulate their own goals in important areas of life (Williams et al., 2000). Indeed, people may have more influence on their own life than they think. Studies have already shown the beneficial effects of both job crafting—where employees actively reframe their work physically, cognitively, and socially (e.g., Wrzesniewski and Dutton, 2001; Demerouti, 2014; Vogt et al., 2016; Wessels et al., 2019)—and leisure crafting (Petrou and Bakker, 2016; Vogel et al., 2016; Petrou et al., 2017). A recent study by Demerouti et al. (2019) suggested that the beneficial implications of job crafting transcend life boundaries, which the authors state have also consequences in terms of experiencing meaning in life.

Building on the above, we suggest that the conscious process of “life crafting” could be similarly beneficial in helping people to find fulfillment and happiness (see Berg et al., 2010; Schippers, 2017). Importantly, life crafting is related to the most important areas of life, and thus allows for a more holistic approach in terms of shaping one’s life. We formally define life crafting as: *a process in which people actively reflect on their present and future life, set goals for important areas of life—social, career, and leisure time—and, if required, make concrete plans and undertake actions to change these areas in a way that is more congruent with their values and wishes.*

The process of life crafting fits with positive psychology and specifically the salutogenesis framework, which states that the extent to which people view their life as having positive influence on their health, explains why people in stressful situations stay well and may even be able to improve adaptive coping (Antonovsky, 1996). Salutogenesis focuses on factors that can support health, well-being, and happiness, as opposed to factors that cause disease (pathogenesis). The salutogenetic model with its’ central element “sense of coherence” is concerned with relationships around health, stress, and coping (Johnson, 2004). In his approach, Antonovsky views health and illness as a continuum, rather than a dichotomy (Langeland et al., 2007). Importantly, the framework assumes that people have resources available (biological, material, and psychosocial) that enable them to construct coherent life experiences (Mittelmark et al., 2017). The idea of salutogenesis is also closely tied to the literature on human flourishing that states that health defined as the absence of illness or disease does not do justice to what it means to be well and thriving (Ryff and Singer, 2000). Broaden-and-build theory can be used to make sense of how this may work out in practice: if people imagine a better future, they will be on the lookout for resources, because they have developed a more positive and optimistic mindset (Fredrickson, 2001; Meevissen et al., 2011). Over time, this broader mindset helps them to acquire more skills and resources and this may in turn lead to better health, happiness, and performance (Garland et al., 2010). When people have a purpose in life and are more balanced, this may have positive ripple effects on the people around them (Barsade, 2002; Quinn, 2005; Quinn and Quinn, 2009). Recent research suggests that health benefits of having stronger purpose in life are attributable to focused attention to and engagement in healthier behaviors (Kang et al., 2019). Indeed, stronger purpose in life is associated with greater likelihood of using preventative health services and better health outcomes (Kim et al., 2014). Importantly, the process through which purpose leads to health outcomes seems to be that people with a purpose in life are better able to respond positively to health messages. They showed reduced conflict-related neural activity during health decision-making relevant to longer-term lifestyle changes. Thus, having a purpose in life makes it easier for people to self-regulate (Kang et al., 2019). These results are very promising, as it seems that having a purpose in life can have both mental and physical health benefits, and behavioral interventions to increase purpose in life have been shown to be very cost-effective (e.g., Wilson et al., 2019). Importantly, purpose in life by writing about personal goals has been associated with improved academic performance (Morisano et al., 2010; Schippers et al., 2015, 2019; Travers et al., 2015; Schippers, 2017; Locke and Schippers, 2018).

Even so, thinking about how to attain a purpose in life *via* a process of life crafting can raise many questions. These include: what is the best way to set personal, self-congruent goals and start the process of life crafting? How does it work? Does the type of goal matter? Does the act of writing the goals down make a difference? Does it increase resourcefulness, self-efficacy, and self-regulation?

Research suggests that reflecting on and writing down personal goals is especially important in helping people to find purpose and live a fulfilling life (King and Pennebaker, 1996; King, 2001), and that in general writing sessions longer than 15 min have larger effects (Frattaroli, 2006). Indeed, the research on writing about life goals has been noted by Edwin Locke as a very important future development of goal-setting theory (Locke, 2019). Recent research shows that goals need not be specific, as long as plans are, and that writing about life goals and plans in a structured way is especially effective (Locke and Schippers, 2018; for a review see Morisano et al., 2010; Morisano, 2013; Schippers et al., 2015; Travers et al., 2015). As goal-relevant actions may be encouraged by embodied cognition, and embodied cognition has been related to (dynamic) self-regulation, this may be the process through which written goals lead to action (see Balcetis and Cole, 2009). Specifically, through the link between cognition and behavior, it can be seen as beneficial to write down intended actions as this will lay the path to act out the intended actions. The processing of the language facilitates the actions, as it consolidates the imagined actions (Addis et al., 2007; Balcetis and Cole, 2009; Peters et al., 2010; Meevissen et al., 2011). It has been suggested that goal-relevant actions may be encouraged by embodied cognition, through the process of self-regulation (Balcetis and Cole, 2009). Writing about actions one wants to take and very detailed experience in how it would feel to reach those goals, may make it much more likely for people to subtly change their behavior and actions into goal-relevant ones (e.g., looking for opportunities to reach ones goal, thinking more clearly if one wants to spend time on certain activities or not, etc.). Also, the writing can make sure that people realize the gap between actual and desired states regarding goals, and act as a starting point for self-regulatory actions (see King and Pennebaker, 1996). According to Karoly (1993, p. 25), “The processes of self-regulation are initiated when routinized activity is impeded or when goal-directedness is otherwise made salient (e.g., the appearance of a challenge, the failure of habitual action patterns, etc.). Self-regulation may be said to encompass up to five interrelated and iterative component phases of (1) goal selection, (2) goal cognition, (3) directional maintenance, (4) directional change or reprioritization, and (5) goal termination.” We believe that the process of writing about self-concordant goals makes (1) the necessity of goal-directed action salient, (2) starts a process of embodied cognition and dynamic self-regulation, and (3) starts an upward spiral of goal-congruence, goal attainment, and (academic) performance. Dynamic self-regulation is needed in the context of multiple goal pursuits where people manage competing demands on time and resources (Iran-Nejad and Chissom, 1992; Neal et al., 2017). In short, although goals are an important part of any intervention involving life crafting, the intervention and its effects are much broader. Such an intervention may be especially beneficial for college students, as it has been shown that students have lower goal-autonomy than their parents and parents reported higher levels of positive affect, lower levels of negative affect, as well as greater life-satisfaction (Sheldon et al., 2006).

In the interventions to date, which have been mainly conducted with students, individuals write about their envisioned future life and describe how they think they can achieve this life, including their plans for how to overcome obstacles and monitor their goals (i.e., goal attainment plans or GAP; e.g., Schippers et al., 2015). Both goal setting and goal attainment plans have been shown to help people gain a direction or a sense of purpose in life. Research in the area of positive psychology explains that people with a purpose in life live longer, have a better immune system, and perform better, even when one controls for things such as lifestyle, personality, and other factors relating to longevity (for a review see Schippers, 2017). At the same time, it has been suggested that relatively small interventions can have a huge impact on people’s lives (Walton, 2014). Writing about values, passion, and goals is an example of such an intervention, and we claim that having a purpose in life is fundamental and has ripple effects to all areas of life, including health, longevity, self-regulation, engagement, happiness, and performance (Schippers, 2017).

In order to provide a stronger theoretical foundation for this claim, we will describe the development of a comprehensive evidence-based life-crafting intervention that can help people find a purpose in life. The intervention shows very specific actions people can take to fulfill that meaning. We start by assessing existing interventions aimed at setting personal goals and will explore the theoretical and evidence-based foundation for those interventions. After that, we describe what a life-crafting intervention should ideally look like. We end with various recommendations for to how to ensure that many people can profit from this intervention (see also Schippers et al., 2015).

## Ikigai, Meaning in Life, and Life Crafting

The meaning of life used to be an elusive concept for scientists, but in the last couple of years much progress has been made in this area. According to Buettner and Skemp (2016), ikigai—a Japanese term for purpose in life—was one of the reasons why people in certain areas of the world, known as “longevity hotspots,” had such long lives (see also Buettner, 2017). As our medical knowledge of longevity is increasing (e.g., Oeppen and Vaupel, 2002; Menec, 2003; Kontis et al., 2017), so too is our understanding of the associated psychological factors. These days, we have more knowledge of how people can live a meaningful life. Research has shown that ikigai, or purpose in life is related to increases in health and longevity across cultures, sexes, and age groups (Sone et al., 2008; Boyle et al., 2009). This relationship has been found even when things such as lifestyle, positive relationships with others, and general affect were controlled for in the analyses (Hill and Turiano, 2014). Note that, although a purpose in life sounds rather unclear or undefinable, people can derive a purpose in life from many different activities. It has been found that these activities can range from volunteering to giving social support to the elderly or even taking care of pets, and all of these have been shown to be related to an increase in happiness, better health outcomes, and greater longevity (for a review see McKnight and Kashdan, 2009). Indeed, in a study of 43,391 Japanese adults, it was found that, over a seven-year follow-up period, mortality was lower among those subjects who indicated that they had found a sense of ikigai or purpose in life (see also Sone et al., 2008; Schippers, 2017). Research among Japanese students has shown that enjoyable and effortful leisure pursuits can enhance student’s perception of ikigai. Ikigai was defined by the authors as “the subjective perceptions that one’s daily life is worth living and that it is full of energy and motivation” (Kono et al., 2019). They also found that leisure activity participation, general satisfaction with leisure activities, and the positive evaluation of leisure experiences were related to higher perception of ikigai (Kono, 2018; Kono and Walker, 2019). (Martela and Steger, 2016) suggested that meaning in life has three components: coherence, purpose, and significance. They state that “meaning in life necessarily involves (1) people feeling that their lives matter, (2) making sense of their lives, and (3) determining a broader purpose for their lives” (Martela and Steger, 2016). Also, Heintzelman et al. (2013) note that there are numerous positive physical and mental outcomes associated with self-reported meaning in life, such as health, occupational adjustment, adaptive coping, lower incidence of psychological disorders, slower age-related cognitive decline, and decreased mortality. Both the theory of ikigai and salutogenesis stress the coherence and purpose part, and other researchers have also picked up on these important elements (e.g., Urry et al., 2004; Martela and Steger, 2016). A review by Martela and Steger (2016) distinguished coherence, purpose, and significance as defining elements of meaning in life. Relatedly, theorizing around ikigai has shown that a sense of coherence develops around three distinct mechanisms, (1) valued experiences, (2) authentic relationships, and (3) directionality (Kono, 2018).

Practically, the importance of happiness to cultures and nations across the world has been indicated clearly by the value placed on it by the United Nations (UN). In 2012, UN Secretary-General Ban Ki-moon commissioned the first World Happiness Report, ranking countries according to people’s level of happiness. The UN’s 2016 Sustainable Development Goals Report included the goal of ensuring sustainable social and economic progress worldwide. In the UN’s 2017 happiness report, “eudaimonia,” a sense of meaning or purpose in life similar to ikigai, is mentioned as an important factor. This is based on research showing the importance of eudaimonic well-being. Indeed a review of research on hedonic and eudaimonic well-being concluded that autonomy and the integration of goals are important predictors of vitality and health (Ryan and Deci, 2001; Huppert et al., 2004) see also (Ryff, 2014). Self-determination theory, a macro theory of human motivation and personality, proposes that only self-endorsed goals will enhance well-being (Ryan and Deci, 2000). This pattern of findings is congruent with the examples we started with (i.e., the self-endorsed goals of Schwarzenegger and the heteronomous goals of Brian) and has also been supported in cross-cultural research, showing that the autonomy of goal pursuit matters in collectivistic and individualistic cultures, and for males and females (Hayamizu, 1997; Vallerand et al., 1997; Chirkov and Ryan, 2001; Ryan and Deci, 2001). As Ryan and Deci (2001, p. 161) conclude: “It is clear that, as individuals pursue aims they find satisfying or pleasurable, they may create conditions that make more formidable the attainment of well-being by others. An important issue, therefore, concerns the extent to which factors that foster individual well-being can be aligned or made congruent with factors that facilitate wellness at collective or global levels.”

The above shows that finding a purpose in life can have far-reaching consequences for individual happiness and performance but also for the well-being and happiness of people around them (Ryan and Deci, 2001). However, finding a purpose in life often requires a lengthy search, and some people never manage to find purpose in life (Schippers, 2017). The developments in terms of ensuring people find their true passion and at the same time help make the world a better place coincide with exciting developments in the area of social psychology. Positive psychology, or the scientific study of human flourishing that aims to optimize human functioning within communities and organizations, has become very influential both within and outside the scientific community (Gable and Haidt, 2005; Donaldson et al., 2015; Al Taher, 2019). It should be noted, however, that this area of study has also faced some criticism, as positive psychology behaviors such as forgiveness may not be functional in all contexts and circumstances (McNulty and Fincham, 2012). Nevertheless, several studies have shown that human flourishing is related to mental and physical health (e.g., Park et al., 2016), and reviews and meta-analyses have shown that positive psychology interventions work in terms of improving well-being and (academic) performance (Sin and Lyubomirsky, 2009; Durlak et al., 2011; Mongrain and Anselmo-Matthews, 2012; Waters, 2012). Thus, making sure that people receive positive psychology interventions, especially those relating to purpose in life, seems a viable and inexpensive way to help millions of people to have a better and healthier life (Menec, 2003; Seligman et al., 2005). Personal goal setting and life crafting seem the best way forward in this respect.

## Values, Passion, and Personal Goal Setting

Life choices can be seen as crucial turning points in someone’s existence. Yet, most people find it difficult to make such important decisions. In particular, young adults struggle with the important life decisions they are expected to make as they move into early adulthood (Sloan, 2018). Recent research has shown that people with a purpose in life are less likely to experience regulatory issues during health decision-making and find it easier to make positive health-related lifestyle decisions (Kang et al., 2019), and it may be especially important to find a purpose in life for young adults (Schippers, 2017). Without such a purpose in life, a lot of time and energy is often “fretted away” on social media and on “busyness,” for instance (Bruch and Ghoshal, 2002, 2004; for a review see Schippers and Hogenes, 2011). At the same time, many people complain of having a lack of time, and it seems that it is more and more important to make conscious decisions on what to spend time on (Menzies, 2005). Life crafting using a personal goal setting intervention seems an important prerequisite in making these decisions. While in the past goal-setting theory has always stressed the importance of specific measurable goals (Locke and Latham, 2002), the act of writing about personal goals seems to be effective by defining very broad goals and linking these to specific goal-attainment plans. Research on the act of writing about personal goals started with Pennebaker’s research on traumatic writing (Pennebaker, 1997; Pennebaker and Chung, 2011). It was shown that writing about traumatic events was related to a decrease in depression and an increase in mental health (Gortner et al., 2006; Pennebaker and Chung, 2011). King (2001) suggested that future-oriented writing about one’s “best possible self” has a similar positive effect on an individual’s well-being, without the short-lived negative effect on mood that occurred after writing about traumatic events. Indeed, it has been shown that imagining one’s best possible self increases optimism and lowers depression (for a meta-analysis see Peters et al., 2010; Malouff and Schutte, 2017). Oyserman et al. (2006) found that a brief intervention that connected the positive “academic possible selves” of low-income minority high-school students with specific goal-attainment strategies improved their grades, standardized test scores, and moods.

Viktor Frankl, an Austrian neurologist and psychiatrist who had survived the holocaust, used his experience to formulate a theory on the meaning of life. He concluded that life can have meaning even in the most impoverished circumstances (Frankl, 1985, 2014). This is interesting, since this also means that good conditions are not an absolute prerequisite for formulating a goal in life. In contrast, it seems that having a goal in life can make people more resilient in terms of surviving harsh conditions. Wong (2014) described the logotherapy developed by Frankl as consisting of five testable hypotheses, including the self-transcendence hypothesis, the ultimate meaning hypothesis, and the meaning mindset hypothesis. These predict among other things that belief in the intrinsic meaning and value of life, regardless of circumstances contributes to well-being, and that a “meaning mindset,” as compared to a “success mindset,” leads to greater eudaimonic happiness and resilience (Wong, 2014). While this is important in terms of knowing what works for well-being and happiness, when people do not have a clear sense of purpose in life or know what they value in life and why, writing down their thoughts and formulating a strategy for their life is important. That does not have to be a lengthy process, but spending a few hours every couple of years might be enough (and is more than most people do).

People who keep searching for meaning without finding it, or who have conflicting goals, are often dissatisfied with themselves and their relationships (Steger et al., 2009). It is quite natural that in earlier stages of their life, people are often still searching for a sense of purpose or meaning in life. However, as stated before, later in life the search for meaning is related to lower levels of well-being (Steger et al., 2009). There is some evidence that having a sense of purpose is associated with organized goal structures and pursuit of goals and provides centrality in a person’s identity (Emmons, 1999; McKnight and Kashdan, 2009). It is thus important that people start thinking about their purpose in life as early as possible and repeat this process at all stages of life when they feel they should readdress their goals, such as when going to college, starting a new job, etc.

### Warding Off Anxiety and Having a Fulfilling Life—Two Side of the Same Coin?

Another line of research has focused on the role of purpose as a protective mechanism against various types of psychological threat, such as mortality salience, or the awareness of an individual that death is inevitable, causing existential anxiety (for a meta-analysis see Burke et al., 2010). These are anxiety-provoking experiences and are common for most people. Ways of coping include having a purpose in life and striving for and accomplishing goals as well as strengthening close relationships (Pyszczynski et al., 2004; Hart, 2014). In line with this, research in the area of terror management has shown that self-esteem as well as a worldview that renders existence meaningful, coherent and permanent buffers against existential anxiety resulting from mortality salience (Burke et al., 2010; Pyszczynski et al., 2015). Indeed, death reflection, a cognitive state in which people put their life in context and contemplate about meaning and purpose, as well as review how others will perceive them after they have passed (Cozzolino et al., 2004), has been proposed as an important prerequisite for prosocial motivation sometimes influencing career decisions (Grant and Wade-Benzoni, 2009). Reducing anxiety and living a fulfilling and meaningful life are two sides of the same coin, since having a purpose in life gives people the idea that their life will continue to have meaning, even after their death (Ryan and Deci, 2004; McKnight and Kashdan, 2009).

### The Science of Wise Interventions

Starting with the work of Kurt Lewin (e.g., Lewin, 1938), and after decades of research and testing, we now have a much better sense of what works and what does not in terms of psychological interventions. Most of these interventions aim to change behavior and improve people’s lives. In general, these work by changing people’s outlook on life: by giving them a sense of purpose. This is the basis of most interventions that also deal with coping with stressors and life transitions, for instance. Goal setting with the aim of formulating a purpose in life is one of the psychology’s most powerful interventions, and it has been shown that even a short and seemingly simple intervention can have profound effects (Wilson, 2011; Walton, 2014). In his review, Walton (2014) describes the “new science of wise interventions”: precise interventions aimed at altering specific psychological processes that contribute to major social problems or prevent people from flourishing. These “wise” interventions are capable of producing significant benefits and do so over time (Walton, 2014). These interventions are “psychologically precise, often brief, and often aim to alter self-reinforcing processes that unfold over time and, thus, to improve people’s outcomes in diverse circumstances and long into the future” (Walton, 2014, p. 74). Writing down personal goals in a guided writing exercise seems to constitute such an intervention.

### How and Why Does It Work?

Narrative writing has been shown to help people in transition phases cope with life stressors (Pennebaker et al., 1990). Students writing about their thoughts and feelings about entering college showed better health outcomes and improved their grades more significantly than students in a control condition. Also, the experimental group had less home-sickness and anxiety 2–3 months after the writing exercise.

Locke (2019) notes that “…writing about goals in an academic setting for two hours or more would connect with grade goals by implication even if the students did not mention them. The writing process would presumably have motivated them to generalize, to think about what they wanted to achieve in many aspects of their lives and encouraged commitment to purposeful action in more domains than were mentioned” (p. 3). On the same page, he also states that “The above issues could occupy interested researchers for many years.”

Broaden-and-build theory suggests that thinking about an idealized future will be associated with positive thoughts about this future, leading to increased levels of self-regulation, resilience, self-efficacy, and in turn engagement (e.g., Tugade et al., 2004; Tugade and Fredrickson, 2004; Ceja and Navarro, 2009; Fay and Sonnentag, 2012). Self-regulation is defined as “self-generated thoughts, feelings, and actions that are planned and cyclically related to the attainment of personal goals” (Boekaerts et al., 2005, p. 14). Many authors contend that goal setting enhances self-regulation and agree that this is the mechanism by which goals are related to action (Latham and Locke, 1991; Oettingen et al., 2000; Hoyle and Sherrill, 2006).

Next to this, the intervention itself may be a form of embodied writing, an act of embodiment, entwining in words our senses with the senses of the world (Anderson, 2001), stimulating what has been written down to act out in real life. However, theorizing around embodied writing and the act of writing as a form of embodied cognition is still in an embryonic stage. Especially research around the effect on writing on our daily actions is lacking in evidence. There is plenty of evidence that these small, written interventions have an effect and can even play a role in redirecting people (e.g., Wilson, 2011) and that these interventions can have a powerful effects in terms of behavioral change (Yeager and Walton, 2011; Walton, 2014). At the same time, it should be noted that these psychological interventions are powerful but context-dependent tools that should not be seen as quick fixes (Yeager and Walton, 2011). However, in the intervention described in the current paper, people are asked to think about their deepest feelings and motivations and write them down, and embodied cognition may very well play a role in the upward spiral resulting from such an intervention.

## Goal Domain

An important discussion in the literature is whether having a self-serving purpose (*hedonistic*, focused on attainment of pleasure and avoidance of pain) or one that is oriented toward helping others (*eudaimonic*, focused on meaning and self-realization) is more beneficial for happiness (Ryan and Deci, 2001; Keyes et al., 2002). Hedonistic and eudaimonic well-being seem to represent two different kinds of happiness (Kashdan et al., 2008). Although recent research has confirmed that both are related to well-being (Henderson et al., 2013), it is also conceivable that a purely hedonistic lifestyle may be unrelated to psychological well-being in the long run (see Huppert et al., 2004; Anić and Tončić, 2013; Baumeister et al., 2013). According to Schippers (2017, p. 21), “prior research has shown that altruistic goals may be particularly helpful in terms of optimizing happiness. Studies on ‘random acts of kindness’—selfless acts to help or cheer up other people—have shown that these acts strengthen the well-being at least of the person performing that act (Otake et al., 2006; Nelson et al., 2016).” Other research has shown that helping others is better for one’s well-being than giving oneself treats (Nelson et al., 2016). A study by (Steger et al., 2008a) suggested that “doing good” may be an important avenue by which people create meaningful and satisfying lives. Also, it has been found that pursuing happiness through social engagement is related to higher well-being (Ford et al., 2015).

## Toward an Integrated Life-Crafting Intervention

The elements discussed above provide the context for developing a potentially effective life-crafting intervention. Although most agree that describing an ideal vision of the future would be a key element of such an intervention, below we identify other elements that should be included, whether the intervention is designed to improve well-being, happiness, performance, or all of these. According to McKnight and Kashdan (2009), “the creation of goals consistent with one’s purpose may be critical to differentiating between real purpose and illusory purpose” (p. 249). Recent research also showed that it is better to have no calling than an unfulfilled calling (see Berg et al., 2010; Gazica and Spector, 2015), making it also a boundary condition that people follow through on this. The importance of following through was shown in a 15-week study aimed at finding out whether engaging in trait-typical behaviors predicted trait change (Hudson et al., 2018). In this study, students provided self-report ratings of their personality and were required to complete weekly “challenges”—prewritten behavioral goals (e.g., “Before you go to bed, reflect on a positive social experience you had during the day and what you liked about it”). These challenges were aimed at aligning their thoughts, feelings, and behaviors with their desired traits (in case of the example this was extraversion). Importantly, results indicated that the mere acceptance of challenges was unrelated to trait changes. Only actually completing the challenges and performing these behaviors predicted trait change (Hudson et al., 2018). This may also hold true for the intervention described below and may be an important boundary condition. Although we have not found any negative effects of the intervention so far, theoretically it is possible that students formulate an “unanswered calling” which may impact happiness, well-being, and performance negatively. So far, only one study did not find the positive effects of a goal-setting intervention on academic outcomes (Dobronyi et al., 2019). This might indicate that for some groups (in this case economy students) the (brief) intervention is not effective in bringing about behavioral change and increasing academic achievement. Other studies showed a positive effect among management students (Schippers et al., 2015) and self-nominated struggling students (Morisano et al., 2010).

Below we provide broad outlines of one such evidence-based intervention, having first set out in brief the case for this particular intervention. Aligning itself to the UN’s sustainable development goals (SDGs), which relate to economic growth, social inclusion, and environmental protection (Stafford-Smith et al., 2017), Rotterdam School of Management (RSM) changed its mission to being a force for positive change in the world (Rood, 2019). As RSM is educating future leaders, in 2011, it introduced a goal-setting intervention so that first-year students could reflect on their personal goals and values. This is a three-stage intervention. In the first part, students write about their values and wishes as well as their ideal life and the life they wish to avoid, and in the second, they describe their specific goals and goal plans. The third part involves a photoshoot with a professional photographer, where students formulate a statement starting with “I WILL…,” (e.g., I WILL pursue my goal, I WILL inspire and facilitate sustainable development, I WILL create healthier businesses for a healthier world, and I WILL lead by example and inspire others to reach their goals).^1^ This statement and the photo are then put on social media and displayed throughout the school.

The evidence-based goal-setting intervention has had a positive effect on study success, as has been shown by higher academic achievement and decreased dropout rates (Locke et al., 2014; Locke and Schippers, 2018). This was particularly true for ethnic minority and male students, who had underperformed in previous years (Schippers et al., 2015; for an elaborate description of the intervention see the supplementary material). In the meantime, plans have been made to make sure that the intervention is an integral part of the curriculum, so that students will develop skills for self-management and management of others and will consider what impact they can have on the world.

### Elements of the Life-Crafting Intervention

Although developed for students, this intervention could also be useful for people who wish to discover a meaning in life and write down their goals. In the first part of this intervention, people discover what is important to them in all areas of life and write about what they feel passionate about. While this part is aimed at making sure they discover their values and passions, the second part is designed to enable them to put those values and passions into a number of goals and to ensure they formulate plans and back-up plans for achieving those goals (Schippers et al., 2015). In terms of the intervention in this paper, the practical questions that address these issues are shown in section 3 of Table 1.

**Table 1:** Elements and description of a life-crafting intervention.

Elements	Tasks involved
1. Values and passion	Writing about: (1) What they like to do, (2) what kind of relationships they would like to have, both in their private life and their work life, (3) what kind of career they would like to have, and (4) lifestyle choices
2. Current and desired competencies and habits	(1) Qualities they admire in others, (2) competencies they have or would like to acquire, and (3) their own habits they like or dislike
3. Present and future social life	(1) Relationship that energize and de-energize them, (2) kinds of friends and acquaintances that are good for them, (3) kinds of friends and acquaintances they would like to have in the future, and (4) what their ideal family life and broader social life would look like
4. Possible future career (path)	(1) What is important in a job, (2) what is it they like to do, (3) what kind of colleagues do they want, and (4) whom do they want to meet through their work?
5. Ideal versus less ideal future	Best possible self and future when there are no (self-imposed) constraints. Contrast this with “future if no changes are made”
6. Goal attainment and “if-then” plans	(1) Formulating, strategizing, and prioritizing goals, (2) identifying and describing ways to overcome obstacles, and (3) monitoring progress toward goals
7. Public commitment to goal	Photo with statement, which communicates their goals to the world; communicating goals to friends, co-workers

#### Discovering Values and Passion

Discovering one’s passion has two sides: Doing what you “like” is often said to be important, but it seems that discovering what you find “important” is more helpful in igniting passion, as this is more values-based and will contribute to self-concordance (Sheldon and Houser-Marko, 2001; Ryff and Singer, 2008). Recent research (e.g., Jachimowicz et al., 2017) has shown that it is important that people pursue a career that is in line with what they find to be “important,” rather than engaging in activities that they “like”; it found that those who engaged in activities that they liked (feelings-oriented mindset) exhibited less passion than those who engaged in activities that they thought were important (values-oriented mindset). Thus, while it is important that people discover what they feel passionate about, ideally this passion should also be aligned with values that they hold dear, such as collaboration, equality, and honesty (Sheldon, 2002).

There is, however, also a difference between harmonious and obsessive passion (for a meta-analysis, see Vallerand et al., 2003; Curran et al., 2015). People with an obsessive work passion experience more conflict between work and other areas of life, and work is more related to their self-worth (Vallerand et al., 2003). Harmonious passion was shown to be related to positive outcomes such as flow and enhanced performance, whereas obsessive passion was related more to negative outcomes, such as excessive rumination and decreased vitality (Curran et al., 2015). Discovering a (harmonious) passion is not always easy.

In a life-crafting intervention, questions on this area could be similar to those listed in section 1 of Table 1, involving also life style choices. In particular, choosing a lifestyle that involves physical activity seems to be a powerful way not only to increase self-regulation and self-control (for a review see Baumeister et al., 2006; Oaten and Cheng, 2006), but also to prevent mental illness, foster positive emotions, buffer individuals against the stresses of life, and help people thrive when they have experienced adversity (Faulkner et al., 2015, p. 207).

#### Gap Between Current Versus Future State: Current and Desired Competencies and Habits

In order to achieve a match between values and passion, it is important to become aware of one’s current habits and competencies as a first step in changing/adapting (cf., Schippers et al., 2014). Being aware of the habits you would like to change is important in promoting positive behavioral change (Holland et al., 2006; Graybiel and Smith, 2014). Since most of our daily behavior is habitual, and this is usually functional in that it allows us to perform many tasks with minimum cognitive effort, but this same mechanism also makes habits hard to break (Jager, 2003). Being aware of our habits and reflecting on them can be a first step in breaking them (Schippers and Hogenes, 2011; Schippers et al., 2014); implementation intentions (i.e., if-then plans: “If situation Y is encountered, then I will initiate goal-directed behavior X!”) have also been shown to help in breaking old habits and forming new ones (Holland et al., 2006). Many people have habits they would like to change (relating, for example, to eating behaviors, physical health, or substance use). However, it has been shown that the effect of good intentions such as New Year’s resolutions is very minimal (Marlatt and Kaplan, 1972; Pope et al., 2014) and that it is the extent to which people have self-concordant goals, coupled with implementation intentions, that leads to successful changes in behavior (Mischel, 1996; Koestner et al., 2002). Self-concordant goals are personal goals that are pursued out of intrinsic interest and are also congruent with people’s identity. Research has shown that if people pursue goals because they align with their own values and interests, rather than because others urge them to pursue them, they typically exhibit greater well-being (Sheldon and Houser-Marko, 2001). This was shown to be true across many cultures (Sheldon et al., 2004). In a life-crafting intervention, questions on this area could be similar to those listed in section 2 of Table 1.

#### Present and Future Social Life

Research shows that people with a strong social network live longer and are healthier and happier (Demir et al., 2015; Haslam et al., 2016). This network does not necessarily have to be very big, and it seems that, as one grows older, the quality of the relationships in this network becomes more important than the quantity (Carmichael et al., 2015). Recent research places more emphasis on the quality of relationships, specifically showing that quality in terms of the social and emotional dimensions of relationships is related to mental well-being (Hyland et al., 2019). The quality of the network has also been shown to be helpful during a transition to college (Pittman and Richmond, 2008). Although at first sight it may seem odd to think about what kind of acquaintances and friends one would like to have, it may pay off to think about this carefully. Certain kinds of relationships, so called high-maintenance relationships, require a lot of time and energy (Schippers and Hogenes, 2011; Fedigan, 2017) and often are characterized by negative interactions that can even influence self-regulation (Finkel et al., 2006). It seems important that in general people seek out interaction with others who are supportive and from which they receive energy rather than those that cost energy. In a life-crafting intervention, questions on this area could be similar to those listed in section 3 of Table 1. Practical questions in the intervention in this respect could be: think about your current friends and acquaintances. What kind of relationships energize you? What kind of relationships require energy? Why is that? What kind of friends and acquaintances do you need? What kind of friends and acquaintances would you like to have in the future? What does your ideal family life and broader social life look like?

#### Future Life: Career

Work is an important part of life. For many it is important to have a job that suits them, and a job which they feel passionate about and from which they can get energy (see Werner et al., 2016; Downes et al., 2017). However, research on mental illness prevails the literature in occupational health psychology, despite a call for a shift toward more research into positive psychology as antipode for work-related health problems such as job burnout. Especially in times where employees are required to be proactive and responsible for their own professional development, and to commit to high quality performance standards, it is important to think about activities that energize people and make them feel engaged with their work (Bakker et al., 2008; Schippers and Hogenes, 2011). Relatedly, research on job crafting shows that people can actively enhance the personal meaning of their work and make it more enjoyable by changing cognitive, task, or relational aspects to shape interactions and relationships with others at work (Wrzesniewski and Dutton, 2001). Consequently, it is not always the job itself but the meaning you give to it that is important (Demerouti et al., 2015). It is also important to think about when and where you do each particular task, in order to manage your daily energy (Wessels et al., 2019).

It should be noted, however, that it is also important to see work in relation to other areas of life. Christensen (2010) noted that many of his contemporaries ended up working 70-h working weeks and also were often divorced and estranged from their children over time. They could not imagine that this end result was a deliberate choice, so it seems important to choose the kind of person you want to become not only in your career but also in other areas of life (Christensen, 2010). This also means making strategic decisions about how to allocate your time and energy, instead of letting daily hassles make these decisions for you (Christensen, 2017). In a life-crafting intervention, participants could be asked to think about what they would ideally like to do in their job, and what kinds of people they might be working with, either directly or indirectly. They could be asked to reflect on their education and their career, and to consider what they feel to be important in a job and what their ideal colleagues would be like. The questions would thus be similar in nature to those shown in section 4 of Table 1.

Of course, some people choose a job that they do not necessarily like a lot but then make sure their leisure time is filled with meaningful activities (Berg et al., 2010), and leisure crafting has been shown to make up to a certain extent for having few opportunities for job crafting. So weighing up the balance between work life and leisure activities and making conscious decisions in this respect seems very important.

#### Key Element: Ideal Future Versus Future If You Do Not Take Action

As people are able to think about and fantasize a future (Oettingen et al., 2018), it is key that the future they envisage is one that is attractive to them. Likewise it is vital they formulate plans of how to achieve their desired future (implementation intentions) and contrast this in their minds with an undesired future (Oettingen and Gollwitzer, 2010; Oettingen et al., 2013). In a university context, and more generally in order to stay engaged, it is important that people choose goals that are self-concordant. It has been shown that if people formulate such goals implicitly by visualizing their best possible self, this can be very powerful and has a stronger effect on well-being than exercises such as gratitude letters (Sheldon and Lyubomirsky, 2006). Other research has shown that writing about the best possible self in three domains—personal, relational, and professional—leads to increased optimism (Meevissen et al., 2011). A meta-analysis showed that best possible self was a particularly powerful intervention in terms of enhancing optimism (Malouff and Schutte, 2017). If this optimism is also turned into concrete plans for the future, there is an increased chance that this positive envisioned future will become a reality (cf., Schippers et al., 2015).

Based on the theorizing above, it should be stressed that in the intervention students formulate goals that *they* find important, not ones that others (parents, peers, or friends) find important or that are pursued solely for reasons of status. In the instructions in the intervention, the students are advised to choose goals that *they* think are important and want to pursue and not to choose goals that others (parents, peers, and friends) think are important. Otherwise, they will live someone else’s life. In order to make sure that they do not choose goals that will be detrimental to themselves or others, they are also advised to not describe an ideal life that includes harming themselves or others.

Additionally, it is also important that people imagine the future they are likely to face if *they do not do anything*. This represents a goal-framing effect, or the finding that people are more likely to take action when they are confronted with the possible consequences of not doing so (Tversky and Kahneman, 1981). It might be useful to ask participants to visualize both a desirable and an undesirable future and to get them to contrast the two (see Oettingen, 2012; Brodersen and Oettingen, 2017). This would be a form of “metacognitive self-regulatory strategy of goal pursuit” (Duckworth et al., 2013, p. 745; cf. Schippers et al., 2013; see also Schippers et al., 2015). Other research has shown that positive “deliberate mental time travel” (or MTT) was related to a significant increase in happiness but not when the MTT was negative or neutral. However, neutral MTT was related to a reduction in stress (Quoidbach et al., 2009). In the intervention (see also Table 1, section 5), participants are asked what their future would look like if they did not change anything. What would their life look like 5–10 years down the road?

#### Goal Attainment Plans

After finishing the elements as described above, it is important for intervention participants to formulate concrete goals and plans. In the meta-analysis undertaken by Koestner et al. (2002), it was concluded that it is important for personal goal setting to be combined with if-then plans. Self-concordance—the feeling that people pursue goals because they fit with their own values and interests—and goal attainment plans are important for goal progress (Locke and Schippers, 2018). Since the rewards that come from achieving a significant life goal are often attained in the future, it is important to formulate concrete goals and also to identify the small steps toward them (see Trope and Liberman, 2003). While the first part of the student intervention is aimed at discovering their passions and ideas about their ideal life, the second part is much more concrete and follows the steps set out in research on goal setting, SMART goals, and if-then plans (Oettingen et al., 2013, 2018). The idea is that by making concrete plans and identifying obstacles (if-then plans), people are better able to visualize their desired future and will be less tempted to engage in activities that distract them from their goal (Mischel, 1996; Mischel and Ayduk, 2004).

In this part of the intervention, ideally any obstacles to the plans will also be identified. In addition to the research on mental contrasting, which generally indicates that one should visualize both the goal and the obstacles to it (e.g., Sevincer et al., 2017), it is important that one should also visualize a way of overcoming those obstacles. This may be a vital element, as research has shown that mental contrasting works best for people who are very confident about succeeding (Sevincer et al., 2017). The elements are outlined in Table 1, section 6. The idea is that, based on what participants write when describing their ideal future, they then identify a number of goals (usually about six to eight), which could be personal, career, and/or social goals (e.g., Morisano et al., 2010; Schippers et al., 2015; Locke and Schippers, 2018). As detailed implementation plans have been shown to aid progress toward goals (Gollwitzer, 1996), it is vital for participants to set down a detailed strategy for how they will achieve their goals. This part of the intervention asks participants about their motivations for their goals and gets them to consider the personal and social impact of those goals. They should also be asked to identify potential obstacles and how to overcome them and monitor progress toward the goals they have set. Participants should be instructed to be specific and concrete—for instance, to write down things that they will do weekly or daily to further their goals (Morisano et al., 2010; Schippers et al., 2015). It may also be useful to get participants to make a concrete plan of action for the upcoming week and to make them specify for each day the hours they will spend working on the goal they have in mind.

#### Public Commitment

In this part of the intervention, participants can either write down a number of goals and make them public (read them out to others) or have a photo taken to accompany a public (“I WILL…”) statement, as was the case in the RSM intervention (see the examples mentioned earlier). Prior research has found that public commitment can enhance goal attainment (Hollenbeck et al., 1989). This part seems to be related to enhanced commitment to goals as a result of self-presentation (Schienker et al., 1994). Shaun Tomson, a former surfing champion and inspirational speaker, invites audiences to come up with goals and 12 lines, all starting with: “I will…” These lines are spoken aloud in a group as a form of public commitment (Tomson and Moser, 2013). This makes it more likely that people will be more self-regulating toward goal-attainment and will put more effort into reaching their goals, especially if they are highly committed to reaching this goal (McCaul et al., 1987).

## Discussion

Formulating clear goals has been shown to contribute to student well-being and academic success (Morisano et al., 2010; Schippers et al., 2015, 2019; Locke and Schippers, 2018). However, this has been often neglected in education and work settings resulting in a lack of evidence based tools. The effects of goal setting on the well-being of students have hardly been tested. Recently, calls have been made for positive psychology interventions to be made part of the educational curriculum in order to teach students life skills and to combat the rising number of mental health problems such as depression (e.g., Clonan et al., 2004; Seligman et al., 2009; Schippers, 2017).

Informed by the theoretical frameworks of salutogenesis, embodied cognition, dynamic self-regulation, and goal-setting theory, in this paper, we outlined a life-crafting intervention in which participants complete a series of online writing exercises using expressive writing to shape their ideal future. Important elements of such an intervention that were covered are: (1) discovering values and passion, (2) reflecting on current and desired competencies and habits, (3) reflecting on present and future social life and (4) future career, (5) writing about the ideal future, (6) goal attainment plans, and finally (7) public commitment to goals.

The idea is to use the fantasized ideal future to deduce goals and formulate a strategy to reach these goals. Finally, participants commit to their intentions by having a photo taken to accompany their goal statement, which is then made public. We described the key elements of this intervention and outlined the theoretical rationale for each of these elements. As previous research has shown that developing life skills, such as being able to set goals and make plans to achieve them (i.e., goal setting), increases the resilience, well-being, and study success of students (Schippers et al., 2015, 2019; Locke and Schippers, 2018), it may be important to make this intervention available to a wider population.

### Future Research and Developments

As research shows that students in higher education are increasingly experiencing psychological problems, such as depression, anxiety, and burn-out (Gilchrist, 2003; Snyder et al., 2016), an add-on to the goal-setting program as described above is recommended. Rapid developments in the field of artificial intelligence (AI), especially areas such as emotion recognition, natural language processing, and machine learning have great potential to aid students experiencing study-related mental health problems (Kavakli et al., 2012; Oh et al., 2017). For example, a goal-setting exercise could be enhanced by incorporating a digital coach in the form of a goal-setting chatbot. With this type of intervention, students are given immediate, personalized feedback after their writing assignments. After two longer writing assignments, which are part of the curriculum, the chatbot can help students to by asking questions on specific topics (Fulmer et al., 2018). For instance, through personalized questions and feedback the chatbot could stimulate students to regularly reflect on their progress toward reaching a certain goal (“Did I invest enough time into my goals? What could I do to improve this? Which smaller sub-goals could help me to achieve my objective? What obstacles do I face? What ways do I see to overcome them?”). Depending on the answers the chatbot could also provide the students with different strategies. In addition, the chatbot can remind students of their goals and objectives during the year.

The expectation is that this addition to the intervention will allow students to reflect better on their own goals, so that a positive effect on student well-being can be expected and more serious problems can be prevented. What is also innovative is that the chatbot can ask additional questions about the students’ well-being. This gives the chatbot an important role in identifying possible problems. For students who have no problems or whose problems are minor, setting goals and receiving online feedback and coaching will be sufficient. In cases of more severe problems, the chatbot can offer more intensive coaching, or can refer them to the university’s psychological support or other professional services if necessary. In summary, the chatbot could provide a better connection between goal setting and the needs of the individual student and could help to integrate the life-crafting intervention into early stages of students’ academic career and can also deliver mental health care for students. Moreover, it could help integrate the life-crafting intervention with interactional forms of mental health care provided by the chatbot, thereby possibly increasing its effectiveness. In addition, goal diaries might form a way to provide insights into whether students are able to achieve important goals. Such diaries could also be used to assess their level of happiness and well-being and might be easily integrated into the interaction with the chatbot.

Next to examining how promising the intervention is in terms of its effects on students, future research could look at the effects of the life-crafting intervention in organizations. Prior research has shown that the effects from positive psychology interventions in organizations are promising (Meyers et al., 2012). The relationship between different areas of life and decision making with regard to how to spend one’s time seems to be key (Menzies, 2005; Schippers and Hogenes, 2011). Researchers could also examine what role life crafting might play at the team level.

## Conclusion

Despite the obvious upside of experiencing meaning in life and having life goals as described in this paper, many people have difficulty choosing between the seemingly endless number of possibilities. The good news is that it is in principle never too late to find a purpose in life, although recent research suggests that it may be most beneficial to find a direction in life earlier rather than later (see Steger et al., 2009; Bundick, 2011; Hill and Turiano, 2014). It seems that interventions of the kind we have described above may be particularly helpful when one is entering into a new phase of life, such as when starting one’s study or just before entering the job market (see Kashdan and Steger, 2007).

The problem so far has been that most interventions are not easily taken to scale (for an exception see Schippers et al., 2015). Given the relatively low amount of costs and administrative work that the implementation of the outlined life crafting intervention entails, especially when compared to the potential benefits, we recommend its inclusion in student’s curriculums. Getting many (young) people to take part in an online life crafting intervention may be an important step in achieving not only higher academic performance, but also better well-being, happiness, health, and greater longevity (see Schippers et al., 2015). Using technology to assist with life crafting *via* a goal-setting intervention seems to be a particularly promising avenue as this is an approach that can be easily scaled up. Ideally then, these scalable and affordable interventions should not be regarded as an extra-curricular activity; it would be advisable to make them a formal part of the curriculum for all students. In a work context, employees could also benefit as this type of activity might be something that companies could easily offer. In short, life-crafting is about (1) finding out what you stand for (i.e., values and passions), (2) finding out how to make it happen (i.e., goal-attainment plans), and (3) telling someone about your plans (i.e., public commitment). Concluding, it seems that life crafting is about taking control of one’s life and finding purpose. Based on recent findings, it would be well-advised for many of us to carve out time to do an evidence-based life-crafting intervention.

## Author Contributions

MS has written the draft of the manuscript. NZ provided important intellectual input at all stages and helped to develop, review, and revise the manuscript.

### Conflict of Interest

The authors declare that the research was conducted in the absence of any commercial or financial relationships that could be construed as a potential conflict of interest.

## Footnotes

^1^see https://www.rsm.nl/iwilleveryone/. A student, participating in the intervention, described its effect on him as follows (see also Singeling, 2017). “I studied, or at least I attempted to study, a lot of different things before I came here. But usually I stopped halfway through. And then I ended up here and I liked the courses well enough, but once again it was completely unplanned. I came here because, well, it was expected of me to finish some kind of university course. *When I got here, and all the “I WILL” stuff* [life crafting/goal setting] *happened, I thought it was a complete and utter joke. I thought: who needs this kind of stuff? Between the second and the third* [trimester], *so towards the end of the second really, I started to realize that: you know those silly goals I put down? I’m actually close to completing some of those. That got me inspired to apply for the position of mentor for the BA business skills course. And in the third year, for my minor, I took a teaching class. A few of my students who started off basically slacking through everything, they are taking their assignments more seriously. Instead of doing everything the evening beforehand, they are dedicating a week beforehand. It’s tiny steps, but they are tiny steps that would not have happened without the goal setting.* Quite simply, I’m proud of the things that I have been doing, such as teaching, and I’m proud that it came through goal setting. It’s why in the end I have changed my I WILL statement: “I will help the next generation to be better.” From this extract, it can be seen that the intervention seemed to inspire the student to be clearer about his goals, to dedicate time to them, and also to use them to help other students. Furthermore, it serves to illustrate the concept of an upward spiral (Sheldon and Houser-Marko, 2001; Sekerka et al., 2012), where trough tiny steps (starting to study for an exam earlier) goals are attained.
